# Effects of Thickening Agents Used in Dysphagia on the In Vitro Dissolution of Gliclazide

**DOI:** 10.3390/pharmacy14020044

**Published:** 2026-03-04

**Authors:** Ayman Allahham, Seerat Fatima, Ieva Stupans, Thilini Thrimawithana, Vivek B. Nooney

**Affiliations:** Pharmacy, School of Health and Biomedical Sciences, STEM College, Bundoora West Campus, RMIT University, Bundoora, VIC 3083, Australiaieva.stupans@rmit.edu.au (I.S.); thilini.thrimawithana@rmit.edu.au (T.T.); vivek.nooney@rmit.edu.au (V.B.N.)

**Keywords:** dysphagia, gliclazide, thickening agents, in vitro dissolution, dissolution kinetics

## Abstract

Dysphagia is common among older adults and frequently necessitates the use of thickening agents to facilitate safe swallowing of medicines, which may in turn alter their bioavailability. This study investigated the impact of two commercially available lubricants—Gloup^®^ Forte and extremely thick water—on the in vitro dissolution behaviour of immediate-release gliclazide tablets. Dissolution studies were conducted using crushed and whole tablets in different media consisting of reverse osmosis water, phosphate buffer (pH 6.8), and 0.1 N HCl at 37 °C. Dissolution profiles were compared using similarity factor (*f*_2_) analysis and modelled using established kinetic equations. Gliclazide dissolution was significantly delayed in the presence of Gloup^®^ Forte across all media for both crushed and whole tablets, with *f*_2_ values below 50, indicating dissimilar profiles. Dissolution kinetics confirmed that mixing the formulated gliclazide with Gloup^®^ Forte delayed the release and reduced the dissolution rate constant for drug from both crushed and whole gliclazide tablets in media studied.

## 1. Introduction

Dysphagia, defined as difficulty in swallowing, is a significant health concern, particularly among elderly individuals and those with chronic illnesses who experience some degree of dysphagia [1]. This condition is commonly associated with neurological disorders such as stroke, traumatic brain injury, Parkinson’s disease, and Alzheimer’s disease, all of which can impair the swallowing mechanism and lead to severe complications [2]. Among these complications, the potential impact on medication administration is particularly critical, as oral drugs are used for managing chronic conditions such as diabetes mellitus (DM), a metabolic disorder characterised by persistent hyperglycaemia. The presence of dysphagia can affect the swallowing of these medications, increasing the risk of aspiration pneumonia, and reduction in quality of life [3]. The quality of life could also be impacted as a consequence of altered therapeutic efficacy associated with the medicines’ use.

The prevalence of dysphagia varies across different populations and settings, with higher rates observed among individuals requiring institutionalised care. Studies indicate that approximately 13% of independently living older adults experience dysphagia, while prevalence rates rise to 25% among hospitalised elderly patients and can exceed 60% in high-level nursing facilities [4,5]. Community-based surveys conducted in the Netherlands, Japan, and the United States have reported dysphagia prevalence ranging from 14% to 33% among elderly populations [6,7,8]. In Australia, a population-based study found dysphagia prevalence to be 16%, with the majority of cases involving difficulty swallowing solid foods (54%) or both solids and liquids (38%) [9]. These findings underscore the widespread incidence of dysphagia and highlight the necessity of strategies to enhance medication safety and efficacy in affected individuals.

Thickening agents are commonly employed to improve swallowing safety by modifying the viscosity of liquids for patients with dysphagia. The International Dysphagia Diet Standardization Initiative (IDDSI) developed a globally recognised framework to classify thickened liquids and texture-modified foods across eight levels (0–7), with drinks categorised from levels 0–4 and foods from levels 3–7. Frequently used thickening agents include xanthan gum, tara gum, carrageenan, and maltodextrin, which reduce the risk of aspiration and promote safer swallowing [10]. However, despite their benefits, these agents can alter the pharmacokinetics of oral medications by delaying dissolution and absorption, which may compromise therapeutic outcomes [11,12].

This concern is particularly relevant for antidiabetic medications such as gliclazide. Gliclazide, a second-generation sulfonylurea, lowers blood glucose levels by stimulating pancreatic insulin secretion [13], yet the kinetics of drug release are not understood when administered alongside thickening agents. Many individuals with dysphagia rely on medication modifications, such as crushing tablets and mixing with thickening agents, to facilitate swallowing. However, these alterations can influence drug dissolution and absorption, potentially affecting therapeutic efficacy.

Products such as Gloup^®^ Forte have been marketed as having minimal interactions with medications, with supporting bioavailability studies focusing primarily on paracetamol. Research indicates that the influence of thickening agents on drug release may vary depending on the drug type and formulation [14,15]. However, whether these findings extend to other drugs, including gliclazide, remains uncertain. The limited research examining the impact of thickening agents on these specific medications presents a gap in the literature. Given the essential common role of gliclazide in diabetes management, it is crucial to investigate whether thickening agents influence its dissolution kinetics and possibly its therapeutic effectiveness. While prior studies suggest that thickening agents can impair dissolution [12,16], the extent of this effect on gliclazide has not been thoroughly examined. This study aims to address this knowledge gap and determine whether the claims regarding Gloup^®^ Forte being inert apply to commonly used antidiabetic medications such as gliclazide. In Australia, gliclazide is the most commonly prescribed sulphonylurea.

## 2. Materials and Methods

### 2.1. Materials

APX-Gliclazide tablets (80 mg gliclazide) were purchased from a local community pharmacy. Gliclazide standard material was obtained from Merck (Melbourne, Australia). Two commercial lubricants were used: xanthan/tara gum-based thickened water (IDDSI Level 4, Flavour Creations^®^, Brisbane, Australia, ingredients: water, thickeners (415, 417), sugar, acidity regulator (335), preservative (200)), referred to as extremely thick water, and carrageenan/dextrose-based Gloup^®^ Forte (IDDSI Level 4, Evomed Pty Ltd., Sandy Bay, Australia; ingredients: water, dried glucose syrup, sucrose, maltodextrin, carrageenan, potassium sorbate, citric acid, flavour). These were selected as they are commonly used as medication lubricants and also showed a significant effect on metformin dissolution. The viscosity of Flavour Creations^®^ is 1.54 Pa·s at 50 s^−1^, whereas the viscosity of Gloup^®^ Forte is 1.07 Pa·s at 50 s^−1^ [16]. Reverse osmosis (RO) water (Milli-Q^®^ EQ 7000, Sigma-Aldrich, Melbourne, Australia) was used for all dissolution studies. The phosphate buffer (pH 6.8) was prepared by dissolving sodium phosphate tribasic dodecahydrate (Sigma-Aldrich, Australia) in RO water, with pH adjustment to 6.80 ± 0.05 using 0.1 N HCl. The 0.1 N HCl dissolution medium was prepared by diluting concentrated hydrochloric acid (Sigma-Aldrich, Melbourne, Australia) in RO water, with final pH verification. The media were selected as per the United States Pharmacopeia (USP) [17].

All dissolution media were filtered through 0.45 μm membrane filters prior to use and were maintained at 37.0 ± 0.5 °C. pH measurements were conducted using a calibrated Seven Direct SD20 pH meter (Mettler-Toledo, Melbourne, Australia) with InLab^®^ Expert Pro-ISM electrode. Media preparation followed United States Pharmacopeia guidelines (USP 43-NF 38).

### 2.2. UV-Spectrophotometric Analysis

Standard solutions of gliclazide (5–25 μg/mL) were prepared in amber volumetric flasks using each dissolution medium (RO water, buffer of pH 6.8 and 0.1 N HCL). Samples were sonicated for 10 min to ensure complete dissolution before analysis. Absorbance was measured at 225 nm using a T60 UV–VIS spectrophotometer (PG Instruments, Leicestershire, UK) with 1 cm quartz cells. All measurements were performed in triplicate, with blank corrections applied for each medium. Linearity for calibration curves in each dissolution media will be checked using the regression coefficient and precision.

### 2.3. In Vitro Dissolution Studies: Crushed Tablets

Gliclazide tablets (APX-Gliclazide, 80 mg) were crushed to a fine powder using a mortar and pestle. The powder was either tested directly or mixed with 10 g of lubricant (Gloup^®^ Forte or extremely thick water) for 10 s before being transferred to dissolution vessels containing 900 mL of media (RO water, buffer of pH 6.8 and 0.1 N HCL solution maintained at 37.0 ± 0.5 °C). The mortar was rinsed with 3 mL of media to ensure complete powder transfer. Dissolution testing was conducted using USP Apparatus 2 (Distek 2500, North Brunswick, NJ, USA) with a paddle speed of 100 rpm. Aliquots (3 mL) were withdrawn at 5, 10, 20, 30, 45, and 60 min using disposable syringes, with immediate blank media replacement. Samples were filtered (0.45 μm), diluted with fresh media to a concentration of 15 μg/mL, and analysed at 225 nm using a T60 UV–VIS spectrophotometer (PG Instruments, UK).

### 2.4. In Vitro Dissolution Studies: Whole Tablets

Dissolution testing for whole APX-Gliclazide (80 mg) tablets was conducted using the same USP Apparatus 2 (Distek 2500, USA) with a paddle speed of 100 rpm. Each vessel contained 900 mL of preheated media (RO water, buffer of pH 6.8 and 0.1 N HCL solution maintained at 37.0 ± 0.5 °C). For experiments with lubricants, tablets were first immersed in 10 g of either extremely thick water or Gloup^®^ Forte, mixed for 10 s to ensure full coating, then transferred to the dissolution vessel. Tablets were placed directly into the vessels. Sampling and analysis followed the same protocol as for crushed tablets.

### 2.5. Similarity Factor (f_2_) Between Dissolution Profiles

Similarity between dissolution profiles was determined using the similarity factor (*f*_2_) calculated with the following formula

(1) $$f_{2}\;=\;50\;\times \;log\left(\frac{100}{\sqrt{1+\;\frac{\sum_{t=1}^{n}{\left(R\left(t\right)-T\left(t\right)\right)}^{2}}{n}}}\right)$$

where *f*_2_ represents the similarity factor, n is the number of time points, R(t) is the mean percentage of reference drug dissolved at time t, and T(t) is the mean percentage of test drug dissolved at time t. This method is widely accepted for comparing dissolution profiles (USP <1092>). An *f*_2_ value > 50 indicates similarity between profiles.

### 2.6. Dissolution Kinetics Using Different Dissolution Equations

To describe the kinetics of the drug release process from tablets (crushed/whole) in all media (RO water, buffer of pH 6.8 and 0.1 N HCL) with/without different medication lubricants (extremely thick water or Gloup^®^ Forte), various kinetic equations were used. These include the zero-order equation, the first-order equation, the Higuchi square root equation, the Hixon–Crowell cube root equation and the Korsmeyer–Peppas equation. The dissolution data were modelled according to the following dissolution equations (Equations (2)–(6)) [18,19]:

Zero-order equation:

(2) $$Q_{t}=Q_{0}+k_{0}t$$

First-order equation:

(3) $$Ln(100-Q_{0})={LnQ}_{0}-k_{1}t$$

Higuchi equation:

(4) $$Q_{t}=k_{H}t$$

Hixon–Crowell cube root equation:

(5) $$\sqrt[{3}]{Q_{0}}-\sqrt[{3}]{Q_{t}}=k_{HC}t$$

Korsmeyer–Peppas equation:

(6) $$Log\left(\frac{Q_{t}}{Q_{\infty }}\right)={Logk}_{K}+nLog\;t$$

where

$Q_{t}:\mathrm{cumulative}\;\mathrm{amount}\;\mathrm{of}\;\mathrm{drug}\;\mathrm{release}$.

$Q_{0}:\mathrm{initial}\;\mathrm{amount}\;\mathrm{of}\;\mathrm{drug}\;\mathrm{release}$.

t: time  of drug release.

$k_{0}:\mathrm{zero}-\mathrm{order}\;\mathrm{release}\;\mathrm{constant}$.

$k_{1}:\mathrm{first}-\mathrm{order}\;\mathrm{release}\;\mathrm{constant}$.

$k_{H}:\mathrm{Higuchi}\;\mathrm{release}\;\mathrm{constant}$.

$k_{HC}:\mathrm{Hixon}-\mathrm{Crowell}\;\mathrm{release}\;\mathrm{constant}$.

$k_{K}:\mathrm{Korsmeyer}-\mathrm{Peppas}\;\mathrm{release}\;\mathrm{constant}$.

$Q_{\infty }:\mathrm{total}\;\mathrm{drug}\;\mathrm{release}\;\mathrm{at}\;\mathrm{infinite}\;\mathrm{time}$.

n:Release component indicating the mechanism.

Dissolution data were modelled with the linear regression model fit using SPSS software (IBM SPSS Statistics 29, USA) using the above equations. Discrimination between these models was determined using the following statistical parameters: the coefficients of determination (R^2^), adjusted R^2^, Akaike Information Criterion (AIC) which is an approximately unbiased estimator of the expected Kullback–Leibler information of a fitted model, which can be used as a discrepancy measure between the actual and the fitted model, RSS which is the residual sum of squares, and RMSE which is the root mean square error. R^2^ and adjusted R^2^ closer to 1 and lower AIC, RSS and RMSS indicate a better fit of the model. The best model then applied to determine the release kinetics and its mechanism.

### 2.7. Data Analysis

The statistical significance of the difference between dissolution profiles (percentage of dissolved drug at a selected time) or dissolution parameters calculated from the selected kinetic modelling was determined with one-way ANOVA using Tukey’s post hoc test calculated in IBM SPSS Statistics software (v.29). *p* values of <0.05 were accepted as statistically significant.

## 3. Results

### 3.1. UV-Spectrometric Calibration Curves

The Beer’s law standard curves for gliclazide in different media (RO water, pH 6.8 buffer, and 0.1 N HCl) were determined at 225 nm over five concentrations (5, 10, 15, 20 and 25 μg/mL) in triplicate (Table 1). The results showed no deviation from linearity with regression coefficients (R^2^) of 0.9997 (water), 0.9999 (pH 6.8 buffer), and 0.9995 (0.1 N HCl). Precision in measured absorbance values at all concentrations was demonstrated with less than 1% standard deviation for all dissolution media. Method validation confirmed limits of detection (LOD) of 0.5 μg/mL, with precision <1% RSD. Quality control samples (mid-range concentrations) were analysed every 10 samples to ensure consistency.

### 3.2. In Vitro Dissolution of Crushed APX-Gliclazide Tablets

**Figure 1:**
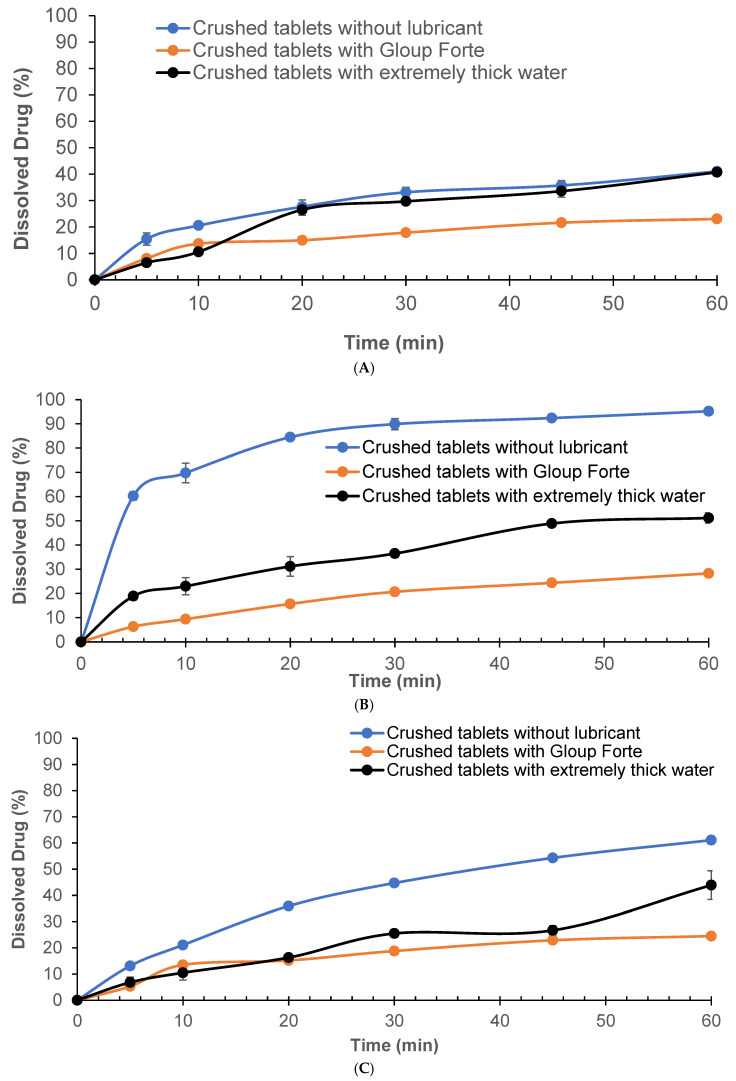
Effect of lubricants on the in vitro dissolution of gliclazide from crushed tablets with/without different lubricants (Gloup^®^ Forte and extremely thick water) in different buffer media ((**A**) RO water, (**B**) buffer of pH 6.8, and (**C**) 0.1 N HCL solution) measured using a Distek dissolution apparatus 2500 at 37.5 °C.

The dissolution behaviour of crushed APX-Gliclazide tablets both alone and following incorporation into Gloup^®^ Forte or extremely thick water is shown in Figure 1A, B and C in different dissolution media (phosphate buffer RO water, buffer pH 6.8 and 0.1 N HCl), respectively. All data represent mean values ± standard deviation (n = 5).

Dissolution of crushed tablets mixed with Gloup^®^ Forte in RO water exhibited the slowest release at 23.0% compared to the control group (without lubricant), which demonstrated the fastest dissolution of 41.0% at 60 min, while tablets mixed with extremely thick water reached 40.7%, nearly matching the control (Figure 1A). Statistical analysis showed that Gloup^®^ Forte significantly reduced dissolution compared to both the control and extremely thick water (*p* < 0.001). In phosphate buffer, gliclazide released from crushed tablets without any lubricant reached 95.2% by 60 min, indicating rapid dissolution (Figure 1B). When Gloup^®^ Forte was added, the dissolution was markedly reduced to only 28.3% of drug released after 60 min. Extremely thick water also slowed the release, though to a lesser extent, achieving 51.2% at the same time point (60 min). Significant differences were observed between the control (without lubricant) and both lubricants conditions (*p* < 0.001). When tested in 0.1 N HCl (Figure 1C), the trend remained consistent. Crushed tablets without lubricants achieved 61.1% dissolution at 60 min, while the tablets mixed with extremely thick water reached only 43.9%, and those mixed with Gloup^®^ Forte released only 24.5% of the drug, reinforcing the same effect of lubricants on the dissolution of gliclazide from crushed tablets in all media studied. The presence of Gloup^®^ Forte significantly slowed the dissolution rate compared to both the control and extremely thick water (*p* < 0.001). Notably, the dissolution performance of crushed tablets mixed with Gloup^®^ Forte did not significantly differ between pH 6.8 and 0.1 N HCl (*p* = 1.000), suggesting limited pH dependence. However, extremely thick water showed improved dissolution in acidic conditions compared to pH 6.8 (*p* = 0.02), indicating a potential interaction between formulation properties and medium acidity.

### 3.3. In Vitro Dissolution of Whole APX-Gliclazide Tablets

**Figure 2:**
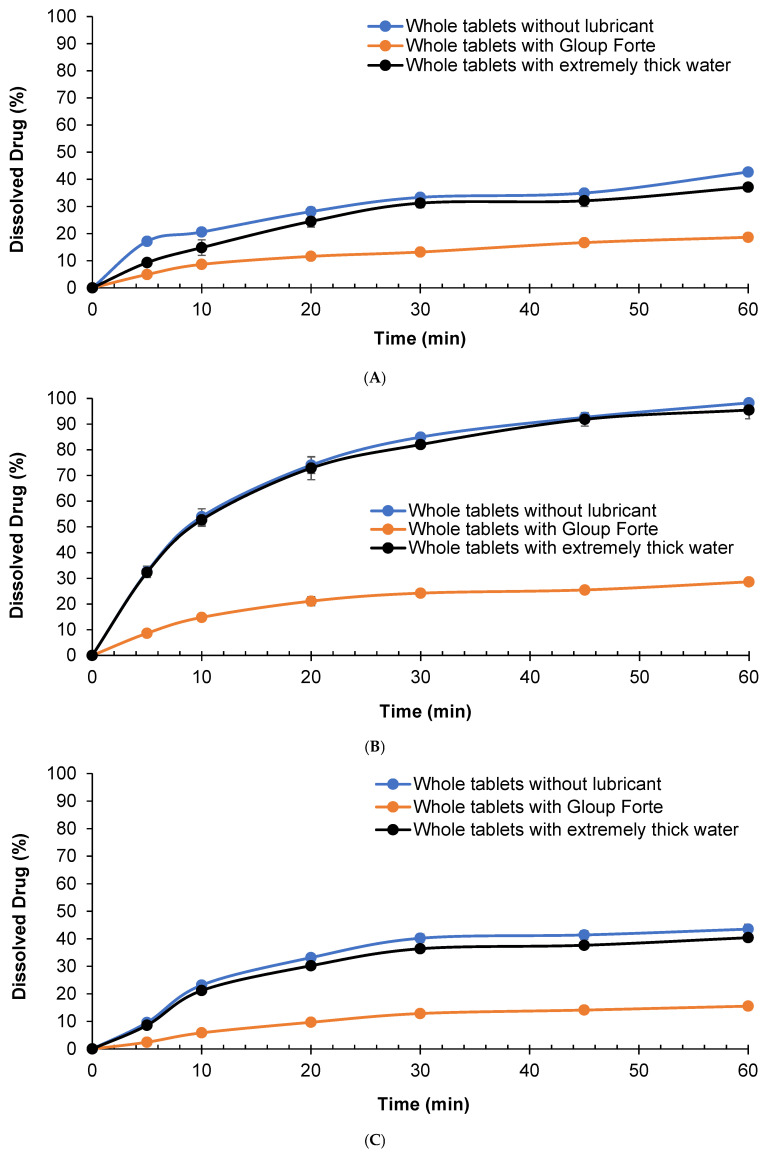
Effect of lubricants on the in vitro dissolution of gliclazide from whole tablets with/without different lubricants (Gloup^®^ Forte and extremely thick water) in different buffer media ((**A**) RO water, (**B**) buffer of pH 6.8, and (**C**) 0.1 N HCL solution) measured using a Distek dissolution apparatus 2500 at 37.5 °C.

The dissolution behaviour of whole APX-Gliclazide tablets was assessed in three different media (phosphate buffer RO water, buffer pH 6.8 and 0.1 N HCl) with and without the addition of the lubricants Gloup^®^ Forte or extremely thick water and is shown in Figure 2.

The results indicated that mixing Gloup^®^ Forte with gliclazide tablets slowed the dissolution rate, reaching only 18.6%, 28.7% and 15.5% compared with 42.7%, 98.3% and 43.5% after 60 min of dissolution in water, buffer pH = 6.8 and 0.1 N HCl, respectively (*p* < 0.001 for all comparisons with the control tablets mixed with Gloup^®^ Forte). These results confirmed that Gloup^®^ Forte significantly delayed the drug release of gliclazide from whole tablets in all media. Whole tablets mixed with extremely thick water showed a similar dissolution rate to the control although the percentage of drug dissolved at 60 min was statistically different in water and 0.1 N HCl (*p* < 0.001 and *p* = 0.04, respectively) but not statistically different in buffer pH = 6.8 (*p* = 0.088).

Taken together, these findings demonstrated that Gloup^®^ Forte consistently impeded gliclazide dissolution across all media, with the most pronounced effect in buffered and acidic environments.

### 3.4. Comparison of In Vitro Dissolution Profiles Using Similarity Factor (f_2_)

**Figure 3:**
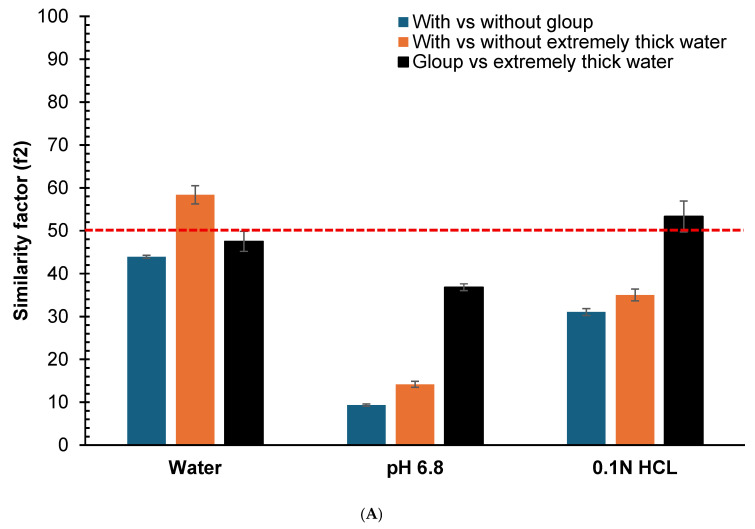

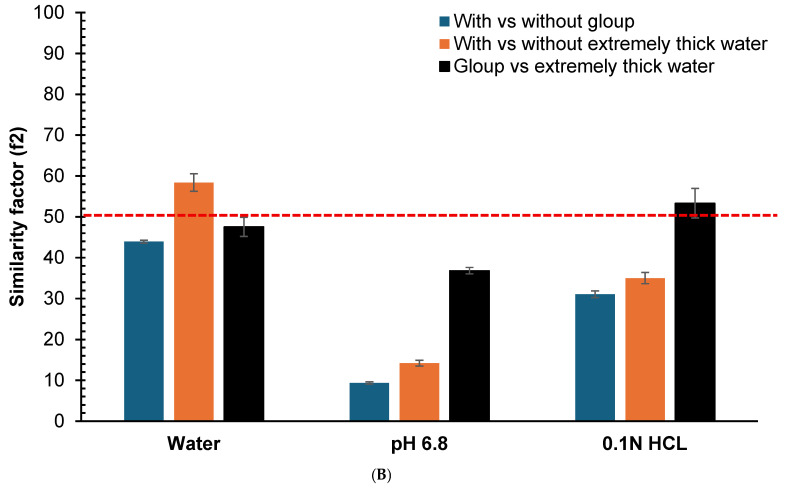
Similarity factor (*f*_2_) comparison calculated from the dissolution profiles at different time points (5, 10, 20, 30, 45 and 60 min) for the vitro dissolution studies of (**A**) crushed gliclazide and (**B**) whole gliclazide tablets with/without different lubricants (Gloup^®^ Forte and extremely thick water) using different media (RO water, buffer of pH 6.8 and 0.1 N HCL solution) conducted using a Distek dissolution apparatus 2500 at 37.5 °C. The red dashed line indicate the 50 cutoff value for determining similarity.

The similarity factors (*f*_2_) comparing dissolution profiles for crushed and whole tablets are presented in Figure 3. For crushed gliclazide tablets (Figure 3A), the *f*_2_ values for Gloup^®^ Forte versus control were 43.9 (SD = 0.4), 9.4 (SD = 0.3) and 31.1 (SD = 0.8) in water, buffer pH = 6.8 and 0.1 N HCl, respectively, with all *f*_2_ values below 50 indicating dissimilar profiles. For extremely thick water versus control, the *f*_2_ values were 58.4 (SD = 2.1), 14.2 (SD = 0.7) and 35.0 (SD = 1.4) in water, buffer pH = 6.8 and 0.1 N HCl, respectively, indicating similar profiles in water (*f*_2_ > 50) but dissimilar profiles in buffer pH = 6.8 and 0.1 N HCl (*f*_2_ < 50).

For whole tablets (Figure 3B), Gloup^®^ Forte versus control showed *f*_2_ values of 37.5 (SD = 0.5), 13.1 (SD = 0.2) and 31.7 (SD = 0.7) in water, buffer pH = 6.8 and 0.1 N HCl, respectively, with all *f*_2_ values below 50 indicating dissimilar profiles. For extremely thick water versus control, the *f*_2_ values were 62.0 (SD = 4.1), 71.2 (SD = 6.0) and 70.4 (SD = 3.0) in water, buffer pH = 6.8 and 0.1 N HCl, respectively, indicating similar profiles in all media (*f*_2_ > 50).

These results quantitatively confirm that Gloup^®^ Forte causes greater deviation from control dissolution profiles in all media and compared with extremely thick water. The deviation in dissolution also depends on whether crushed or whole gliclazide tablets were used. The effect was seen with crushed gliclazide tablets (and not whole tablets) mixed in buffered or acidic media, but not with water.

### 3.5. Results for Dissolution Kinetics Using Different Dissolution Equations

**Figure 4:**
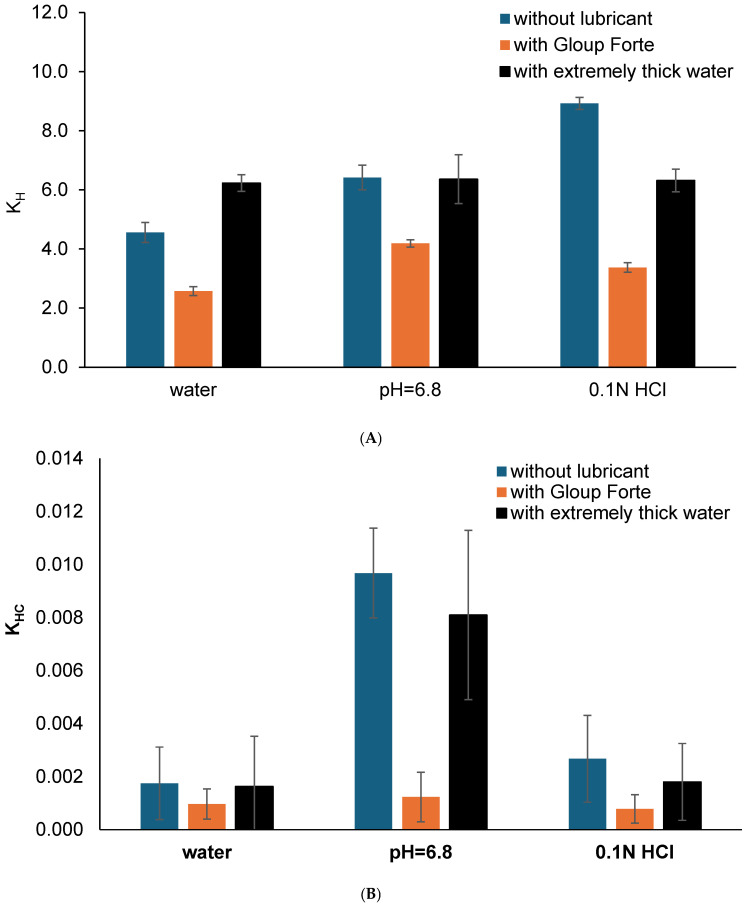
(**A**) The Higuchi release constant (kH) and (**B**) the Hixson–Crowell rate constant (kHC) calculated from the Higuchi kinetic dissolution modelling (using linear regression of model fit from IBM SPSS) for the vitro dissolution of gliclazide from crushed tablets mixed with/without medication lubricants (Gloup^®^ Forte and extremely thick water) in different buffer media (RO water, buffer of pH 6.8 and 0.1 N HCL solution) conducted using a Distek dissolution apparatus 2500 at 37.5 °C.

The dissolution data of gliclazide from tablets mixed with/without different lubricants (extremely thick water and Gloup^®^ Forte) in different buffer media (A: RO water; B: buffer of pH 6.8; C: 0.1 N HCL) was modelled using dissolution kinetic equations (zero order, first order, Higuchi, Hixon–Crowell and Korsmeyer–Peppas) and the comparison of the goodness of fit parameters is shown in Appendix A and Appendix B for crushed and whole tablets, respectively. The data demonstrate that Higuchi and the Hixon–Crowell models may be used to evaluate the impact of the swallowing aid on the dissolution rate. Figure 4A,B show the dissolution rate constants obtained from the Higuchi and the Hixon–Crowell models for crushed gliclazide tablets.

The results indicated that the release rate constant (k_H_) for gliclazide from crushed tablets ranged from 2.6 to 8.9 and showed a decrease in the Higuchi dissolution rate constant k_H_ when these tablets were mixed with Gloup^®^ Forte in all media (*p* < 0.001 for all comparisons without lubricant or with extremely thick water). On the other hand, the Hixon–Crowell dissolution rate constant (k_HC_) for gliclazide from whole tablets showed a similar trend of decrease in k_HC_ when these tablets were mixed with Gloup^®^ Forte in buffer with pH = 6.8 only (*p* < 0.001) but not in water or 0.1 N HCl (*p* > 0.682). This could be due to the variability and large standard deviations from the calculation of k_HC_ as it ranged from 0.00057 to 0.00968.

## 4. Discussion

This study investigated the influence of two commercially available lubricants (Gloup^®^ Forte and extremely thick water) on the in vitro dissolution of gliclazide tablets, using three biorelevant media (RO water, pH 6.8 buffer and 0.1 N HCl). Consistent with previous findings using metformin tablets [16], Gloup^®^ Forte significantly slowed drug release in all media, whereas extremely thick water had a markedly lesser effect. Dissolution is substantially reduced for gliclazide from the tablets, whereby whole tablets dissolved to only 28.7% with Gloup^®^ Forte in pH 6.8 buffer, compared to 98.3% in the absence of lubricant. This aligns with previous findings that increased viscosity may reduce drug diffusion from the tablet surface, delaying dissolution [11]. In contrast, extremely thick water, despite its higher relative viscosity, had minimal impact on the dissolution of gliclazide in water and pH 6.8 buffer. For example, gliclazide tablets dissolved to 95.5% with extremely thick water, nearly identical to controls (98.3%) in a buffer of pH 6.8 compared to 28.7% dissolution for the gliclazide tablets when mixed with Gloup^®^ Forte. These results suggest that viscosity alone does not fully account for the effect on dissolution. The type and structure of thickening agent appear to play a more critical role than absolute viscosity. Prior studies have similarly shown that highly viscous substances like jam, which has been used as a swallowing aid, can variably affect drug dissolution, depending on gelling agents such as pectin and fruit solids [20]. Extremely thick water, which incorporates xanthan gum, forms a more uniform and rigid structure [21], likely contributing to its more predictable and limited impact on drug release.

The results with gliclazide tablets established the trend that Gloup^®^ Forte delayed dissolution significantly in both whole and crushed tablet forms across all dissolution media, with the most pronounced effect in acidic conditions. In 0.1 N HCl, crushed gliclazide tablets reached only 24.5% dissolution with Gloup^®^ Forte compared to 61.1% without any lubricant. Extremely thick water, while still slowing dissolution (43.9%), remained less inhibitory than Gloup^®^ Forte. This suggests that the interaction between Gloup^®^ Forte and the drug is stronger and less dependent on medium pH. The differences between the two agents likely arise from their composition. Gloup^®^ Forte contains carrageenan and maltodextrin, which can form a gel matrix that entraps drug particles. Carrageenan is known for its thermo-reversible gelation and resistance to shear [22,23], features that may contribute to its consistent delay of dissolution across media. In addition, some carrageenan can interact with starch increasing the viscosity. Gliclazide tablets used in the study contain Sodium Starch Glycolate used as an excipient which may interact with the carrageenan polymer, resulting in increased viscosity associated with the microenvironment of particles’, likely leading to observed delays in dissolution (in vitro). Extremely thick water, by contrast, relies on xanthan and tara gum, which do not appear to interact with the drug matrix to alter drug dissolution. This variability suggests that the interaction of extremely thick water with the drug may be more sensitive to changes in ionic strength of the medium or pH, in contrast to Gloup^®^ Forte, which maintained a strong inhibitory effect regardless of the environment. It is interesting to note that both crushed and whole tablets showed similar trends despite the expectation that crushing the tablets may give different penetration and permeation effects. The only exception to this consistent dissolution profile was seen in the acidic media (0.1 N HCl) where the dissolution from whole tablets was significantly slower compared to the crushed tablets in the same media.

Despite manufacturers’ claim that Gloup^®^ Forte does not affect drug absorption, the present data suggest there could be variable drug release following oral administration. The observed delay in drug dissolution, especially under gastric conditions, could have implications for therapeutic efficacy. According to ICH criteria for immediate-release formulations, at least 85% of the drug should be released within 30 min [24]. The Gloup^®^ Forte-mediated delay in dissolution suggests this criterion was not met, particularly in acidic media. Similarity factor (*f*_2_) analysis supported this conclusion, with values below 50 in Gloup^®^ Forte conditions indicating substantial deviation from reference profiles, while extremely thick water generally showed values above 50, suggesting minimal impact.

The rheological behaviour of the agents may further explain the observed outcomes. Agents with higher yield stress, such as Gloup^®^ Forte, resist shear forces more effectively and may persist longer in a semi-solid state during gastric transit [21,25,26]. This limits drug diffusion and can hinder tablet disintegration. Gloup^®^ Forte’s xanthan-based matrix likely contributes to its elastic, high-resistance bolus [27]. We have previously investigated the viscosity of the swallowing aids used in the current study. As reported, the viscosity of extremely thick water was higher (1.54 Pa·s) than that of Gloup^®^ Forte (1.07 Pa·s) (*p* < 0.001) at a swallowing shear rate of about 50 s^−1^; however, this higher viscosity of extremely thick water did not appear to reduce the dissolution rate of metformin from a whole tablet [16]. In another study, extremely thick water, formulated with modified starches, exhibits different gelation and lower yield stress [28], which may explain its weaker impact on drug release. These reports suggest that IDDSI classification may not be sufficient to determine the impact of swallowing aids on medication. It is important to note that in vivo factors—such as feeding state, enzymatic activity, gastric motility, and transit times—were not accounted for in this in vitro study. These physiological variables could also influence drug release and should be considered in future studies [29].

The preferential fit of the Higuchi model for crushed tablets suggests diffusion-controlled release dominates once the tablet matrix is disrupted, whereas the Hixon–Crowell model better describes whole tablets that govern dissolution. The reduction in dissolution rate constants in the presence of Gloup^®^ Forte indicates that this lubricant imposes a layer of resistance. The pH-dependent effect observed for whole tablets further suggests an interaction between the lubricant matrix and the dissolution medium, which may influence drug diffusion processes differently under intestinal versus gastric conditions.

## 5. Conclusions

The use of oral lubricants and thickening agents has become increasingly prevalent in managing dysphagia, particularly among elderly patients and those with chronic conditions such as diabetes. A significant proportion of this population is prescribed immediate-release antidiabetic medications, including gliclazide. This study assessed how commercially available lubricants—specifically Gloup^®^ Forte and extremely thick water—influence the dissolution behaviour of gliclazide tablets under different buffers. The findings indicated that both whole and crushed gliclazide tablets experienced a delay in dissolution when mixed with these agents, with Gloup^®^ Forte producing the most significant reduction across all media tested. This effect appeared linked to the formulation’s rheological properties, such as viscosity and gel-forming capacity. Extremely thick water also slowed drug release, though to a lesser extent, and its impact varied depending on the pH of the medium. These observations are particularly important when considering the pharmacokinetics of immediate-release formulations like gliclazide, where rapid onset of action is required to manage postprandial blood glucose levels. Any delay in dissolution may influence both the timing and extent of absorption, potentially affecting clinical outcomes. While the in vitro model used here cannot fully predict in vivo performance, it strongly suggests a need for caution when co-administering these agents with oral dosage forms in dysphagic patients. This raises concerns for the Quality Use of Medicines (QUM) in patients with dysphagia. The slowed dissolution observed in this study implies that thickening agents—though beneficial for swallowing—may potentially compromise therapeutic efficacy. In particular, Gloup^®^ Forte’s consistent effect on impeding dissolution across different pH conditions highlights its potential to interfere with the kinetics of drug release regardless of the gastrointestinal environment.

Future research should aim to clarify the mechanistic basis of the delayed dissolution and clinical significance of these findings. Further studies aiming to characterise the physicochemical properties of commercially available thickening agents, changes in particle size and the possible chemical/physical interactions between them and selected drugs can explore the mechanism of the observed delay in dissolution. Studies incorporating both in vitro dissolution and in vivo pharmacokinetic data will be essential to determine whether the observed delays in drug release translate into altered therapeutic effects with respect to the onset of action. Additionally, efforts toward the development of new formulations or administration strategies that ensure both safe swallowing and reliable drug absorption are warranted. Until then, healthcare providers should remain vigilant when prescribing medications for patients requiring swallowing aids, especially those with narrow therapeutic windows or time-sensitive pharmacological actions.

## Figures and Tables

**Table 1:** Results for the calibration curve of gliclazide (with concentrations 5, 10, 15, 20 and 25 mcg/mL) in different media (RO water, buffer pH 6.8 and 0.1 N HCl) performed using a T60 UV–VIS spectrometer at 225 nm wavelength (n = 3 for each concentration).

Media	Equation	R^2^
RO water	Absorbance = (0.0385 × concentration) + 0.0107	0.9997
Buffer pH 6.8	Absorbance = (0.0383 × concentration) + 0.004	0.9999
0.1 N HCl	Absorbance = (0.0371 × concentration) − 0.0099	0.9995

## Data Availability

Research data is available and can be requested from corresponding author upon request.

## Appendix A

**Table A1:** The goodness of fit parameters for the in vitro dissolution of gliclazide from crushed tablets mixed with/without medication lubricants (extremely thick water and Gloup^®^ Forte) in different buffer media (RO water; buffer of pH 6.8; 0.1 N HCl) measured using a Distek dissolution apparatus 2500 at 37.5 °C and modelled using different dissolution kinetic equations (zero order, first order, Higuchi, Hixon–Crowell and Korsmeyer–Peppas) using linear regression of model fit from IBM SPSS.

Zero order (Qt versus t)									
	RO water			pH = 6.8			0.1 N HCl		
Model parameters	No agent	With Gloup^®^ Forte	With extremely thick water	No agent	With Gloup^®^ Forte	With extremely thick water	No agent	With Gloup^®^ Forte	With extremely thick water
R^2^	0.897	0.88	0.878	0.775	0.942	0.931	0.944	0.862	0.922
Adjusted R^2^	0.893	0.876	0.874	0.767	0.94	0.929	0.942	0.857	0.919
RMSE	3.0	1.8	4.4	6.4	2.0	3.4	4.2	2.6	3.7
RSS	248.5	94.5	552.3	1155.0	112.9	322.1	495.8	186.5	381.1
AIC	67.4	38.4	91.4	113.5	43.8	75.2	88.1	58.8	80.3
First order (Ln (100 − Qt) versus t)									
	RO water			pH = 6.8			0.1 N HCl		
Model parameters	No agent	With Gloup^®^ Forte	With extremely thick water	No agent	With Gloup^®^ Forte	With extremely thick water	No agent	With Gloup^®^ Forte	With extremely thick water
R^2^	0.866	0.816	0.895	0.927	0.870	0.931	0.976	0.764	0.873
Adjusted R^2^	0.861	0.809	0.891	0.924	0.866	0.928	0.975	0.755	0.869
RMSE	0.047	0.033	0.051	0.249	0.036	0.056	0.048	0.039	0.069
RSS	0.063	0.031	0.073	1.730	0.036	0.086	0.065	0.043	0.133
AIC	−181.0	−202.2	−176.6	−81.6	−197.8	−171.6	−180.0	−192.4	−158.6
Higuchi (Qt versus SQR of time)									
	RO water			pH = 6.8			0.1 N HCl		
Model parameters	No agent	With Gloup^®^ Forte	With extremely thick water	No agent	With Gloup^®^ Forte	With extremely thick water	No agent	With Gloup^®^ Forte	With extremely thick water
R^2^	0.913	0.818	0.911	0.953	0.891	0.941	0.985	0.818	0.827
Adjusted R^2^	0.910	0.812	0.908	0.951	0.887	0.939	0.984	0.812	0.821
RMSE	0.038	0.033	0.047	0.199	0.033	0.051	0.039	0.034	0.081
RSS	0.040	0.030	0.062	1.107	0.030	0.073	0.042	0.033	0.182
AIC	−194.6	−203.2	−181.5	−95.0	−203.2	−176.6	−193.1	−200.4	−149.1
Hixon–Crowell (Q_0_^1/3 − Qt^1/3 versus time)									
	RO water			pH = 6.8			0.1 N HCl		
Model parameters	No agent	With Gloup^®^ Forte	With extremely thick water	No agent	With Gloup^®^ Forte	With extremely thick water	No agent	With Gloup^®^ Forte	With extremely thick water
R^2^	0.911	0.889	0.898	0.897	0.952	0.941	0.973	0.878	0.906
Adjusted R^2^	0.908	0.885	0.894	0.893	0.951	0.939	0.972	0.873	0.902
RMSE	0.054	0.031	0.075	0.228	0.032	0.066	0.066	0.043	0.080
RSS	0.080	0.026	0.158	1.461	0.029	0.124	0.122	0.053	0.180
AIC	−173.8	−207.5	−153.4	−86.7	−204.2	−160.7	−161.1	−186.2	−149.5
Korsmeyer–Peppas (Log Qt/Q∞ versus Log time)									
	RO water			pH = 6.8			0.1 N HCl		
Model parameters	No agent	With Gloup^®^ Forte	With extremely thick water	No agent	With Gloup^®^ Forte	With extremely thick water	No agent	With Gloup^®^ Forte	With extremely thick water
R^2^	0.904	0.893	0.928	0.862	0.968	0.939	0.983	0.907	0.923
Adjusted R^2^	0.901	0.895	0.925	0.857	0.967	0.937	0.982	0.904	0.92
RMSE	0.050	0.056	0.082	0.031	0.043	0.041	0.030	0.055	0.069
RSS	0.070	0.086	0.190	0.027	0.052	0.047	0.024	0.086	0.132
AIC	−61.8	−55.6	−31.9	−90.4	−70.7	−73.8	−93.9	−55.6	−42.8

## Appendix B

**Table A2:** The goodness of fit parameters for the dissolution vitro dissolution of gliclazide from whole tablets mixed with/without medication lubricants (extremely thick water and Gloup^®^ Forte) in different buffer media (RO water; buffer of pH 6.8; 0.1 N HCl) measured using a Distek dissolution apparatus 2500 at 37.5 °C and modelled using different dissolution kinetic equations (zero order, first order, Higuchi, Hixon–Crowell and Korsmeyer–Peppas) using linear regression of model fit from IBM SPSS.

Zero order (Qt versus t)									
	RO water			pH = 6.8			0.1 N HCl		
Model parameters	No agent	With Gloup^®^ Forte	With extremely thick water	No agent	With Gloup^®^ Forte	With extremely thick water	No agent	With Gloup^®^ Forte	With extremely thick water
R^2^	0.939	0.914	0.849	0.831	0.82	0.826	0.747	0.86	0.766
Adjusted R^2^	0.936	0.911	0.843	0.825	0.814	0.82	0.737	0.855	0.758
RMSE	2.2	1.4	4.0	9.8	3.0	9.7	6.3	1.8	5.6
RSS	140.1	56.7	454.5	2701.2	257.0	2630.4	1113.4	92.8	870.5
AIC	50.2	23.1	85.5	139.0	68.4	138.2	112.4	37.9	105.0
First order (Ln (100 − Qt) versus t)									
	RO water			pH = 6.8			0.1 N HCl		
Model parameters	No agent	With Gloup^®^ Forte	With extremely thick water	No agent	With Gloup^®^ Forte	With extremely thick water	No agent	With Gloup^®^ Forte	With extremely thick water
R^2^	0.896	0.785	0.840	0.933	0.697	0.843	0.765	0.737	0.781
Adjusted R^2^	0.892	0.777	0.834	0.931	0.687	0.838	0.757	0.728	0.773
RMSE	0.043	0.031	0.058	0.349	0.046	0.467	0.085	0.035	0.074
RSS	0.052	0.027	0.093	3.418	0.060	6.099	0.201	0.034	0.152
AIC	−186.7	−206.4	−169.3	−61.2	−182.4	−43.8	−146.2	−199.5	−154.6
Higuchi (Qt versus SQR of time)									
	RO water			pH = 6.8			0.1 N HCl		
Model parameters	No agent	With Gloup^®^ Forte	With extremely thick water	No agent	With Gloup^®^ Forte	With extremely thick water	No agent	With Gloup^®^ Forte	With extremely thick water
R^2^	0.890	0.799	0.883	0.887	0.786	0.821	0.869	0.757	0.878
Adjusted R^2^	0.887	0.792	0.879	0.883	0.778	0.814	0.865	0.748	0.874
RMSE	0.044	0.030	0.049	0.454	0.039	0.499	0.063	0.034	0.055
RSS	0.054	0.026	0.067	5.784	0.043	6.976	0.112	0.032	0.084
AIC	−185.6	−207.5	−179.1	−45.4	−192.4	−39.8	−163.7	−201.3	−172.3
Hixon–Crowell (Q_0_^1/3 − Qt^1/3 versus time)									
	RO water			pH = 6.8			0.1 N HCl		
Model parameters	No agent	With Gloup^®^ Forte	With extremely thick water	No agent	With Gloup^®^ Forte	With extremely thick water	No agent	With Gloup^®^ Forte	With extremely thick water
R^2^	0.948	0.921	0.865	0.969	0.834	0.918	0.773	0.867	0.793
Adjusted R^2^	0.946	0.918	0.860	0.968	0.828	0.915	0.765	0.862	0.785
RMSE	0.040	0.023	0.070	0.180	0.052	0.269	0.115	0.029	0.099
RSS	0.046	0.015	0.139	0.909	0.075	2.028	0.369	0.024	0.274
AIC	−190.4	−224.0	−157.2	−100.9	−175.7	−76.8	−127.9	−209.9	−136.9
Korsmeyer–Peppas (Log Qt/Q∞ versus Log time)									
	RO water			pH = 6.8			0.1 N HCl		
Model parameters	No agent	With Gloup^®^ Forte	With extremely thick water	No agent	With Gloup^®^ Forte	With extremely thick water	No agent	With Gloup^®^ Forte	With extremely thick water
R^2^	0.933	0.908	0.940	0.921	0.898	0.951	0.855	0.933	0.873
Adjusted R^2^	0.931	0.904	0.938	0.918	0.895	0.949	0.85	0.93	0.868
RMSE	0.036	0.063	0.056	0.050	0.064	0.041	0.086	0.078	0.091
RSS	0.036	0.110	0.089	0.069	0.113	0.046	0.208	0.171	0.232
AIC	−81.8	−48.3	−54.6	−62.2	−47.4	−74.4	−29.1	−35.0	−25.9
